# Acetylation mimic of lysine 280 exacerbates human Tau neurotoxicity *in vivo*

**DOI:** 10.1038/srep22685

**Published:** 2016-03-04

**Authors:** Marianna Karina Gorsky, Sylvie Burnouf, Jacqueline Dols, Eckhard Mandelkow, Linda Partridge

**Affiliations:** 1 Max Planck Institute for Biology of Ageing, Joseph-Stelzmann-Strasse 9b, 50931 Cologne, Germany; CECAD Cologne Excellence Cluster on Cellular Stress Responses in Aging Associated Diseases, 50931 Cologne, Germany; 2 German Center for Neurodegenerative Diseases (DZNE), 53175 Bonn, Germany; CAESAR Research Center, Ludwig-Erhard-Allee 2, 53175 Bonn, Germany; 3 Max-Planck-Institute for Metabolism Research, Hamburg Outstation, c/o DESY, Hamburg

## Abstract

Dysfunction and accumulation of the microtubule-associated human Tau (hTau) protein into intraneuronal aggregates is observed in many neurodegenerative disorders including Alzheimer’s disease (AD). Reversible lysine acetylation has recently emerged as a post-translational modification that may play an important role in the modulation of hTau pathology. Acetylated hTau species have been observed within hTau aggregates in human AD brains and multi-acetylation of hTau *in vitro* regulates its propensity to aggregate. However, whether lysine acetylation at position 280 (K280) modulates hTau-induced toxicity *in vivo* is unknown. We generated new *Drosophila* transgenic models of hTau pathology to evaluate the contribution of K280 acetylation to hTau toxicity, by analysing the respective toxicity of pseudo-acetylated (K280Q) and pseudo-de-acetylated (K280R) mutant forms of hTau. We observed that mis-expression of pseudo-acetylated K280Q-hTau in the adult fly nervous system potently exacerbated fly locomotion defects and photoreceptor neurodegeneration. In addition, modulation of K280 influenced total hTau levels and phosphorylation without changing hTau solubility. Altogether, our results indicate that pseudo-acetylation of the single K280 residue is sufficient to exacerbate hTau neurotoxicity *in vivo*, suggesting that acetylated K280-hTau species contribute to the pathological events leading to neurodegeneration in AD.

Human Tau (hTau) is a natively unfolded, microtubule-associated protein that regulates the assembly and stabilisation of microtubules in neurons. Tau dysfunction is associated with many neurodegenerative diseases including Alzheimer’s disease (AD), the most prominent Tauopathy, which is characterised by both hyperphosphorylated Tau and β-amyloid deposits. However, the mechanisms leading to Tau dysfunction in AD are still not fully understood.

Tau protein is highly post-translationally modified. Given its unstructured nature, it is likely that distinct combinations of post-translational modifications, such as phosphorylation, ubiquitination and acetylation, act in concert, resulting in structural and functional changes that may influence Tau toxicity and function (for review see^1^). Interestingly, next to hTau hyperphosphorylation, reversible lysine acetylation has recently emerged as a modification that may play an important role in the modulation of AD pathology^2^,^3^,^4^,^5^. Indeed, *in vitro* experiments have revealed that the recombinant full-length (FL) hTau protein displays more than 20 putative acetylation sites^2^,^3^,^6^. Interestingly, enhancing FL hTau lysine acetylation through co-incubation with Histone deacetylase 6 (HDAC6) inhibitors influenced hTau phosphorylation^4^, while co-incubation with either CREB-binding protein (CBP) or p300 acetyltransferase enzymes and acetyl-CoA could regulate hTau aggregation^2^,^4^,^6^. In addition, multi-acetylated FL hTau was shown to display a reduced ability to promote tubulin assembly into microtubules *in vitro*^2^.

Immunohistochemical analyses of human AD brains have so far revealed the presence of three acetylated forms of hTau within Tau deposits, namely acetylated-K174-hTau, acetylated-K274-hTau and acetylated-K280-hTau, suggesting a high biological relevance of these modified epitopes in AD pathogenesis^2^,^5^,^7^,^8^. In a recent study, pseudo-acetylation at K174 (K174Q) was reported to result in hTau accumulation and was sufficient to induce cognitive deficits *in vivo* reference 5. However, whether acetylation events at other hTau lysine residues also modulate toxicity *in vivo* is currently unknown. Lysine 280 is of particular interest because its deletion results in hTau aggregation^9^,^10^, indicating a key role of K280 in hTau pathogenicity. Interestingly, in addition to being present in AD brains, acetylated-K280-hTau species were also detected in insoluble fractions from brain lysates of both PS19 and PS19/PDAPP transgenic mouse models of AD and accumulated with age in the cortex of PS19/PDAPP mice, further implying a role for K280 acetylation in hTau aggregation^2^. In addition, the generation of a pseudo-acetylated hTau-K280 mutant using a glutamine substitution has revealed that this residue is important for microtubule bundling in cell culture experiments^2^. Altogether, these experiments suggest a potential role for hTau-K280 in AD pathogenesis. However, whether acetylation of hTau at K280 directly triggers toxicity *in vivo* remains elusive.

The fruit fly *Drosophila melanogaster* has proven to be a powerful model system for the *in vivo* analysis of neurodegenerative diseases^11^,^12^,^13^,^14^. We therefore generated inducible, transgenic fly lines over-expressing the wild-type full length human Tau protein (the 2N4R isoform consisting of 441 amino acids) as well as mutant forms of the latter either mimicking acetylation at lysine 280 with glutamine (K280Q), to model both the charge and chemical structure of acetylated lysine, or abolishing acetylation at this residue while conserving its positive charge, with arginine (K280R)^2^,^3^,^15^,^16^,^17^,^18^. We used on one hand a site-directed integration strategy to ensure comparable expression levels among hTau mutants^19^ so as to unravel in an unbiased way the effect of the single hTau-K280 mutations *in vivo*, and on the other hand an inducible driver system to circumvent any developmental effects potentially related to hTau expression.

Mis-expression of these hTau species in the adult fly nervous system using the pan-neuronal elav-GeneSwitch-Gal4 (elavGS) inducible driver showed that pseudo-acetylated hTau-K280Q significantly increased neurodegeneration and fly locomotion defects. This effect was associated with significant changes in hTau levels and phosphorylation on S262 and T212/S214, but without any overt alteration in hTau solubility. In addition, hTau-K280Q and hTau-K280R species led to a similar reduction of fly lifespan. Altogether, our results suggest that the modulation of the single K280 hTau residue is sufficient to regulate hTau neurotoxicity *in vivo*.

## Results

**Figure 1:**
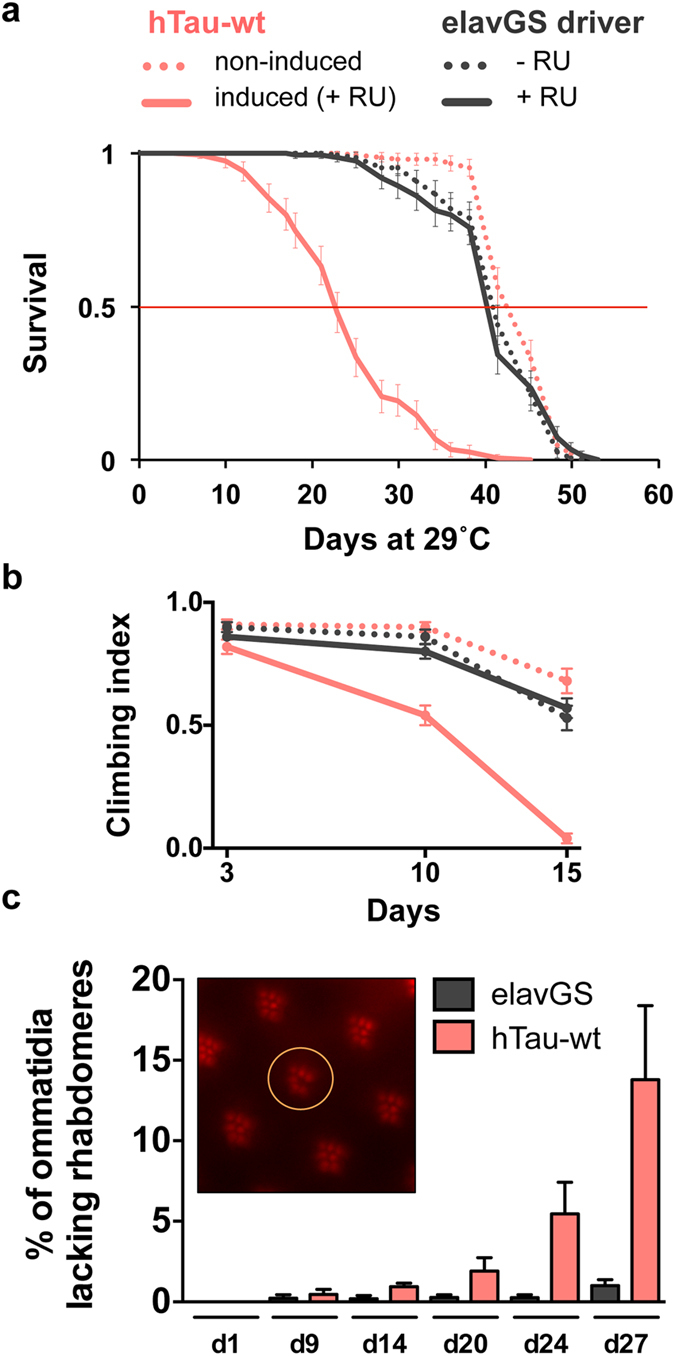
Decreased fly survival, impaired climbing ability and increased photoreceptor neurodegeneration in an adult-onset hTau overexpression model. (**a**) Survival was drastically reduced in flies overexpressing the full-length 2N4R hTau-wt protein (plain vs. dotted red curve) in the adult nervous system (****p < 0.0001, RU486-induced hTau-wt vs. non-induced controls). Survival of the elavGS driver line control was not affected by RU486 feeding (black curves). (**b**) Flies overexpressing the hTau-wt protein (plain red curve) presented progressive, drastic climbing defects as compared to both non-induced controls and elavGS flies (****p < 0.0001, RU486-induced hTau-wt vs. non-induced controls at day 10 and day 15, two-way ANOVA). (**c**) hTau-wt overexpression in adult fly neurons led to progressive photoreceptor neurodegeneration as measured by the percentage of ommatidia lacking rhabdomeres over age (**p < 0.01, one-way ANOVA), while RU486-fed elavGS controls showed no significant neurodegeneration over time (p > 0.05, one-way ANOVA). The inset displays a representative caption of one ommatidium lacking the central rhabdomere (circle), surrounded by several intact ommatidia containing 7 visible rhabdomeres.

To investigate the potential toxicity of adult-restricted neuronal expression of the full-length (2N4R) human Tau protein (hTau-wt) in *Drosophila*, we used the attP/attB targeted integration system together with the UAS-Gal4 binary system to generate a transgenic fly line enabling the standardised and tissue-specific expression of hTau-wt proteins. Adult-onset expression of hTau-wt was achieved by feeding adult flies with the RU486 inducer (Supplementary Figure 1). Interestingly, we observed a drastic alteration of fly survival following homozygous hTau-wt expression in adult neurons using the pan-neuronal elavGS driver (****p < 0.0001 vs. non-induced hTau-wt controls, log-rank test, Fig. 1a), with median lifespans reaching 21.9 and 43.3 days for induced and non-induced hTau-wt transgenic lines, respectively. The toxicity of hTau-wt proteins depended on both transgene copy number (Supplementary Figure 2a) and experimental temperature (Supplementary Figure 2b). The survival of the elavGS driver line control was not affected by RU486 feeding (p > 0.05, Fig. 1a). In line with hTau-wt toxicity on fly survival, we observed striking detrimental effects of hTau-wt expression on fly climbing ability (****p < 0.0001 vs. non-induced controls at day 10 and day 15, two-way ANOVA, Fig. 1b), while the climbing behaviour of elavGS control flies was unchanged upon RU486 feeding (p > 0.05 at all investigated ages, two-way ANOVA, Fig. 1b). We then investigated whether hTau-wt neuronal expression led to neurodegeneration by analysing the loss of photoreceptor neurons in the *Drosophila* compound eye using the quantitative cornea neutralization technique as previously described^12^,^20^. Adult-onset neuronal expression of hTau-wt led to the progressive loss of rhabdomeres in *Drosophila* eyes, with the percentage of affected ommatidia reaching 13.8% ± 4.59% following 27 days of transgene expression (**p < 0.01 along age, one-way ANOVA, Fig. 1c), while the elavGS driver line showed no significant photoreceptor neurodegeneration over time (p > 0.05, one-way ANOVA, Fig. 1c). Altogether, these results indicate significant toxic effects triggered by the adult-onset neuronal expression of hTau-wt in *Drosophila*.

**Figure 2:**
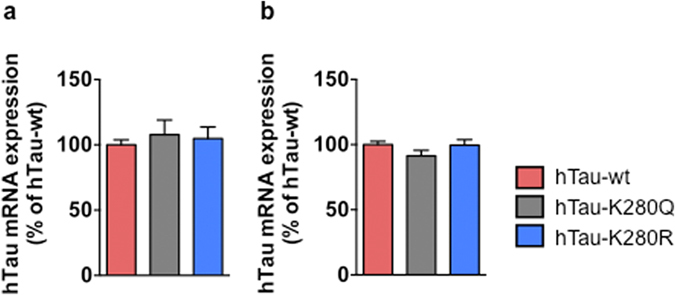
hTau transcript levels. qRT-PCR analysis of hTau mRNA levels in heads of hTau-wt- (red), hTau-K280Q- (grey) and hTau-K280R- (blue) expressing transgenic flies following one (**a**) and five days (**b**) of induction in the adult nervous system (elavGS driver) showed no significant difference among the transgenic lines. p > 0.05, one-way ANOVA, n = 5–6/genotype.

Next, we investigated the influence of hTau acetylation at lysine 280 (K280) on hTau-induced toxicity. Therefore, we generated new transgenic *Drosophila* lines expressing either pseudo-acetylated (K280Q) or pseudo-de-acetylated (K280R) mimic forms of the full-length hTau-wt protein using site-directed mutagenesis. Comparable hTau expression among the transgenic lines was ensured by the use of the attP/attB site-specific integration strategy and verified by qRT-PCR, both shortly after the beginning of transgene expression (p > 0.05, 1 day of RU486 induction, Student’s t-test, Fig. 2a) and following a longer induction period (p > 0.05, 5 days of RU486 induction, Student’s t-test, Fig. 2b).

**Figure 3:**
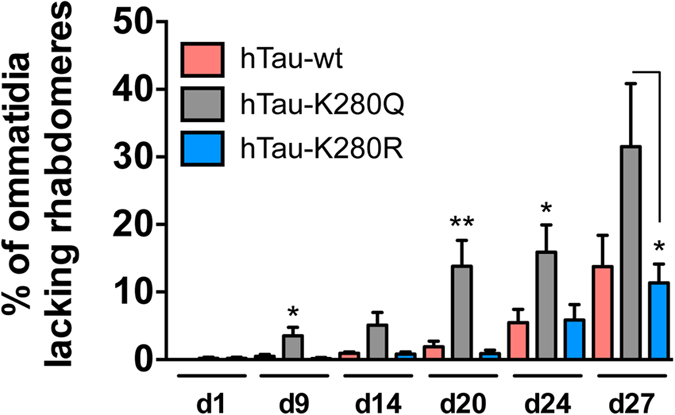
hTau-K280Q expression in adult fly neurons exacerbated photoreceptor neurodegeneration. Quantification of the proportion of ommatidia lacking at least one rhabdomere following the adult-onset expression of hTau-wt (red), hTau-K280Q (grey) and hTau-K280R (blue) in fly neurons (elavGS driver) after 1, 9, 14, 20, 24 and 27 days of induction (Day9 and day24: *p < 0.05, hTau-K280Q vs. hTau-K280R and hTau-wt; day20: **p < 0.01, hTau-K280Q vs. hTau-K280R and hTau-wt; day 27: *p < 0.05, hTau-K280Q vs. hTau-K280R, using one way ANOVA followed by Tukey’s post hoc test).

Then, to evaluate the effect of K280 pseudo-acetylation/de-acetylation on hTau neurotoxicity, we analysed photoreceptor neurodegeneration in the compound eyes of hTau-wt, hTau-K280Q and hTau-K280R transgenic flies. Interestingly, adult-onset neuronal expression of pseudo-acetylated hTau-K280Q species led to a significantly increased photoreceptor neurodegeneration as compared to both hTau-wt and hTau-K280R transgenics, and was detectable as early as following 9 days of transgene expression (*p < 0.05, day 9, one-way ANOVA, Fig. 3). Rhabdomere loss progressively increased with age and affected up to 31.5% ± 9.31% of all ommatidia following 27 days of transgene expression in the hTau-K280Q transgenic line. On the other hand, photoreceptor neurodegeneration measured in hTau-K280R flies compound eyes followed a similar pattern as in hTau-wt transgenics (p > 0.05 at all investigated time points, one-way ANOVA, Fig. 3), while being overall milder than neurodegeneration observed in hTau-K280Q flies (*p < 0.05 and **p < 0.01, one-way ANOVA, Fig. 3). Altogether, these results point to the exacerbated neurotoxicity of hTau species that are acetylated at K280 in the adult fly nervous system.

**Figure 4:**
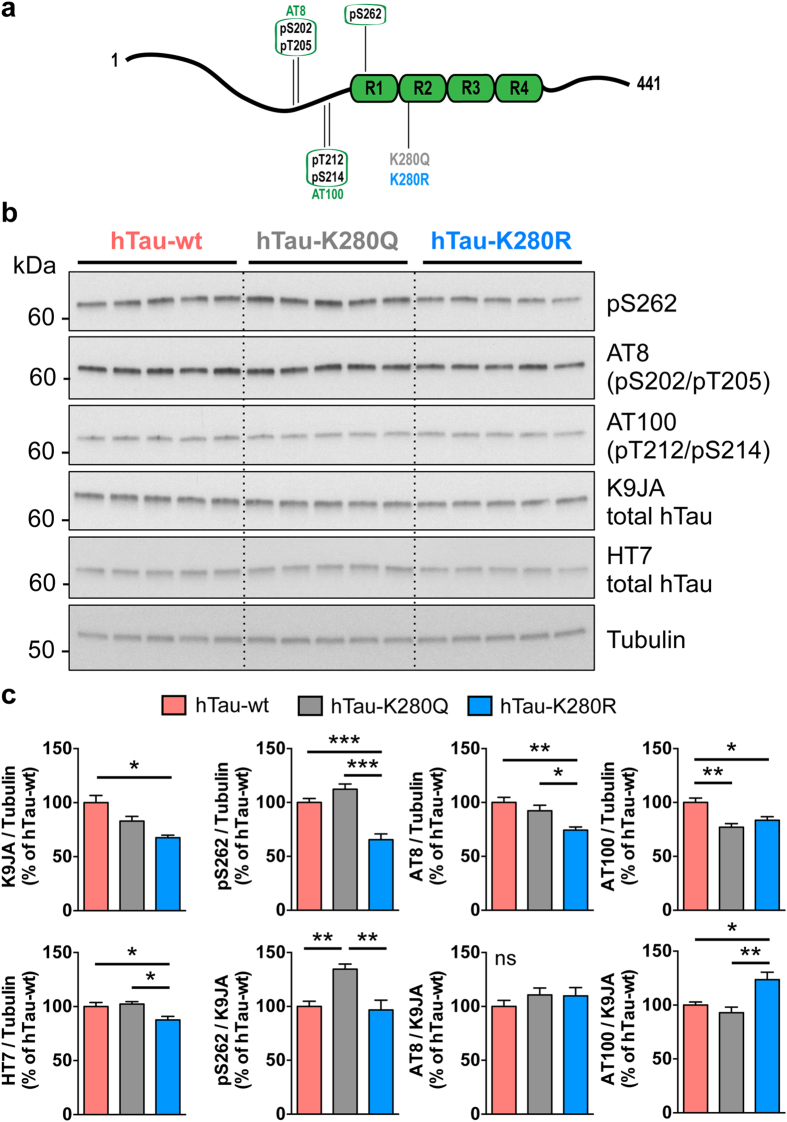
Pseudo-acetylation at K280 influenced the phosphorylation pattern of hTau proteins. (**a**) Illustration of hTau phosphorylation and acetylation sites used in this study. hTau phosphorylation on S262, S202/T205 (AT8) and T212/S214 (AT100) was analysed following pseudo-acetylation or pseudo-deacetylation of hTau at K280 (K280Q and K280R, respectively). (**b**,**c**) Western blot analysis (**b**) and quantification (**c**) of hTau phosphorylation on S262, S202/T205 (AT8) and T212/S214 (AT100) and total hTau levels using the polyclonal K9JA (Dako) and monoclonal HT7 antibodies, following 14 days of hTau expression in the fly nervous system using the elavGS driver. Results are normalised to both Tubulin and total hTau levels and are expressed relative to levels observed in the hTau-wt transgenic line (*p < 0.05, **p < 0.01 and ***p < 0.001, one-way ANOVA followed by Tukey’s post hoc test, n = 5/genotype).

We subsequently investigated whether the differential degree of toxicity induced by these hTau species would be concomitant to an effect on hTau phosphorylation at specific sites (Fig. 4a). Interestingly, we observed that pseudo-acetylation or pseudo-de-acetylation of the single hTau-K280 residue resulted in a significant alteration of hTau phosphorylation as measured in heads extracts from 14-day-old flies. hTau-K280Q mutants showed significantly increased phosphorylation on S262 as compared to both hTau-wt and hTau-K280R flies, when normalised to total hTau (K9JA) levels (**p < 0.01, one-way ANOVA, Fig. 4b,c). Conversely, phosphorylation on the AT100 epitope (pT212/S214) was increased in heads of hTau-K280R flies as compared to hTau-K280Q (**p < 0.01, one-way ANOVA, Fig. 4b,c) and hTau-wt (*p < 0.05, one-way ANOVA, Fig. 4b,c) transgenics. Regarding phosphorylation on the AT8 epitope (pS202/T205), we did not find any difference among our transgenic lines when normalised to total hTau levels (p > 0.05, one-way ANOVA, Fig. 4b,c). These effects of K280 mutation on hTau phosphorylation were also verified in older flies (Supplementary Figure 3).

**Figure 5:**
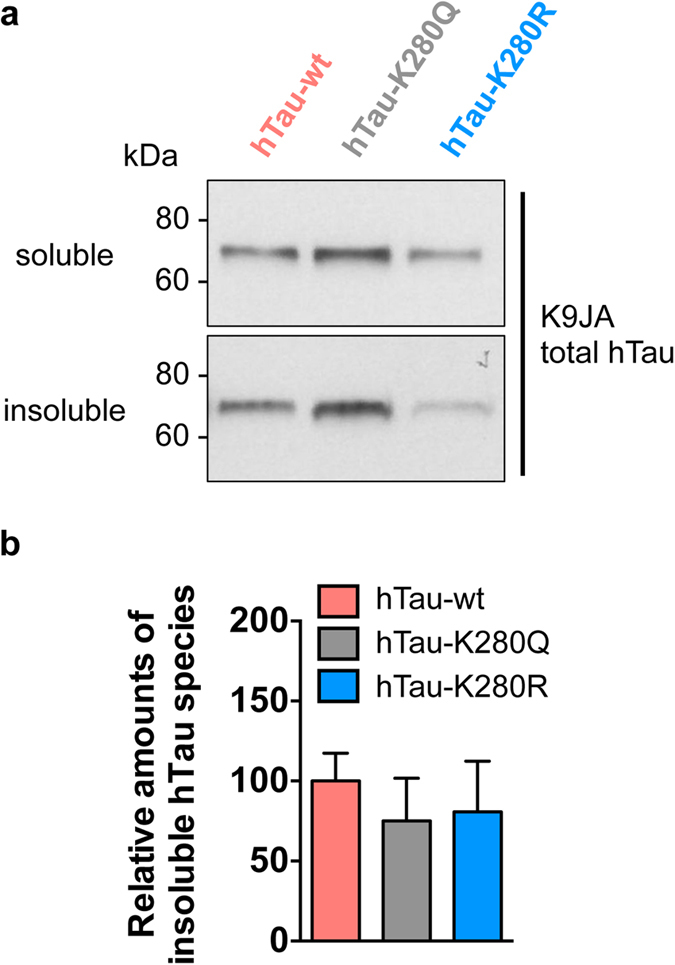
hTau protein solubility was not affected by K280 acetyl-mimic mutation. (**a**) Representative western blots of RAB-soluble and RAB-insoluble/RIPA-1%SDS-soluble hTau fractions retrieved from heads of hTau-wt, hTau-K280Q- and hTau-K280R-expressing flies following 14 days of induction in the nervous system (elavGS driver), using the total hTau K9JA antibody. (**b**) The proportion of insoluble hTau species was quantified and expressed relative to the ratios observed in the hTau-wt transgenic line, p > 0.05, one-way ANOVA. n = 4/genotype.

**Figure 6:**
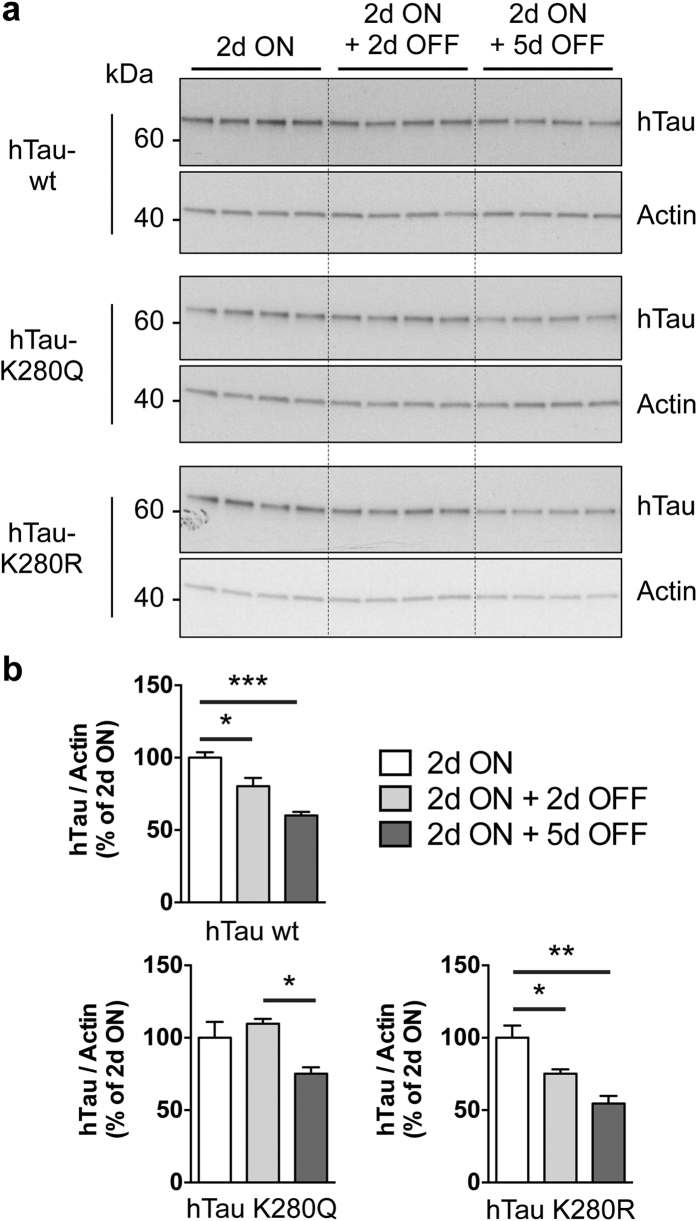
hTau clearance was affected by K280 pseudo-acetylation. Western blot analysis (**a**) and quantification (**b**) of total hTau levels retrieved from adult fly head extracts following neuronal (elavGS-driven) expression of hTau-wt, hTau-K280Q or hTau-K280R following either 2 days of RU486 induction (“2d ON”) or 2 days of RU486 induction followed by exposure to RU486-free food for either 2 or 5 days (“2d ON + 2d OFF” and “2d ON + 5d OFF”, respectively). hTau detection was achieved using the polyclonal K9JA antibody (Dako) and Actin was used for normalisation. Results are expressed relative to levels observed in the “2d ON” condition (*p < 0.05, **p < 0.01 and ***p < 0.001, one-way ANOVA followed by Tukey’s post hoc test, n = 4/condition).

Noteworthy, it appeared that total hTau protein amounts were also regulated by the K280 mutation. Total hTau levels were significantly decreased in the hTau-K280R line using the polyclonal K9JA antibody (*p < 0.05 vs. hTau-wt, one-way ANOVA, Fig. 4b,c) as well as the monoclonal HT7 antibody (*p < 0.05, hTau-K280R vs. hTau-wt and hTau-K280R vs. hTau-K280Q, one-way ANOVA, Fig. 4b,c). Such an effect on total hTau protein levels, occurring despite comparable hTau mRNA levels in all three lines (p > 0.05, one-way ANOVA, Fig. 2), could have been the consequence of a change in hTau solubility. We therefore investigated hTau solubility by means of fractionation using RAB and RIPA-1% SDS buffers in heads of 14-day-old hTau-expressing flies. The vast majority of the hTau pool was retrieved in the RAB-soluble fraction, with a very low proportion of all hTau species being in an insoluble state in hTau transgenic *Drosophila* (Supplementary Figure 4). Notably, we could not observe any overt change in hTau solubility among the transgenic lines using the K9JA anti-hTau antibody (p > 0.05, one-way ANOVA, Fig. 5a,b). Therefore, we used the T22 antibody^21^ to evaluate the formation of soluble hTau oligomers, but we could not find any specific signal in hTau-expressing lines compared to non-transgenic flies (Supplementary Figure 5). These results together suggest that the decreased hTau protein levels observed in the hTau-K280R line (Fig. 4) were likely not due to reduced hTau aggregation. We therefore hypothesized that, alternatively, the degradation of hTau proteins could be differentially regulated in our hTau mutants. We tackled this question by performing a “switch-on/switch-off” experiment, in which flies were exposed to the RU486 inducer for 2 days (“2d ON”) to induce neuronal hTau expression, and then transferred to RU486-free food for either 2 or 5 days (“2d ON + 2d OFF” and “2d ON + 5d OFF”, respectively) to evaluate hTau clearance (Fig. 6). We could observe that, while hTau levels were significantly decreased after 2 days of clearance in both hTau-wt and hTau-K280R transgenic flies (*p < 0.05, “2d ON + 2d OFF” vs. “2d ON”, one-way ANOVA, Fig. 6a,b), we did not highlight any significant clearance of hTau-K280Q proteins at this time point (p > 0.05, “2d ON + 2d OFF” vs. “2d ON”, one-way ANOVA, Fig. 6a,b). Interestingly, hTau levels from all hTau mutants were significantly decreased after 5 days of clearance (Fig. 6a,b), suggesting that clearance of hTau-K280Q species was delayed compared to that of hTau-wt and hTau-K280R, rather than completely halted. We therefore investigated whether the levels of Hsc70 and HSP90, two chaperone proteins involved in protein degradation, were regulated in our hTau transgenic flies. We did not observe any significant difference in their levels in head extracts from 21-day-old hTau-wt, hTau-K280Q and hTau-K280R transgenics (p > 0.05, one-way ANOVA, supplementary Figure 6).

**Figure 7:**
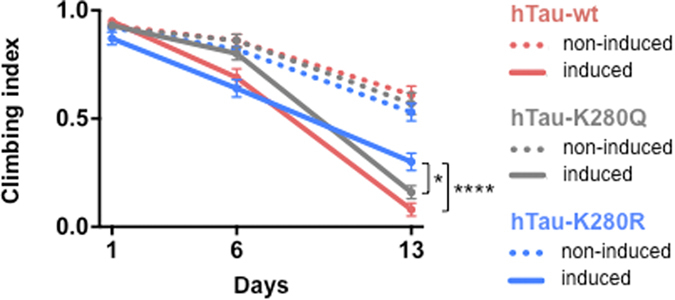
The K280R mutation mitigated hTau-induced climbing defects in old flies. Climbing ability was monitored over time in flies over-expressing either hTau-wt (red), hTau-K280Q (grey) or hTau-K280R (blue) in the adult nervous system (elavGS-driven). Day 13: *p < 0.05, RU486-induced hTau-K280R vs. RU486-induced hTau-K280Q and ****p < 0.0001, RU486-induced hTau-K280R vs. RU486-induced hTau-wt; two-way ANOVA followed by Tukey’s post hoc test.

**Figure 8:**
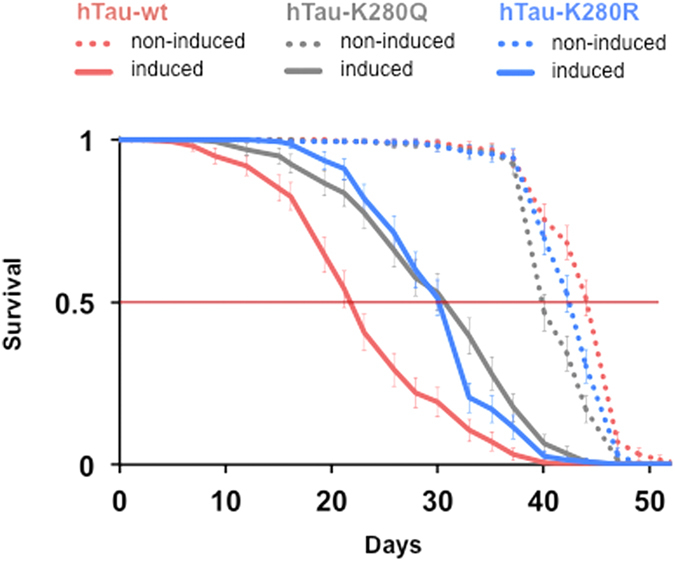
Mutating K280 to a pseudo-acetylated or pseudo-unacetylated state both equally delayed fly death. Representative survival curves of transgenic fly lines expressing (plain curves) either hTau-wt (red), hTau-K280Q (grey) or hTau-K280R (blue) in the adult fly nervous system (elavGS driver and RU486 induction). Survival of non-induced lines is shown as colour-matched dotted curves.

Finally, we evaluated the impact of hTau-K280 acetylation on fly climbing ability (Fig. 7 and supplementary figure 7) and survival (Fig. 8). In line with the neurotoxic effects observed following hTau-K280Q expression in adult fly neurons (Fig. 3), we observed that mimicking the de-acetylated state of lysine 280 in the hTau protein mitigated the toxic effects of hTau expression on climbing ability in older flies (day 13: *p < 0.05, induced hTau-K280R vs. induced hTau-K280Q and ****p < 0.0001, induced hTau-K280R vs. induced hTau-wt; two-way ANOVA, Fig. 7). Though this effect was relatively mild, it was significant and highly reproducible as shown in supplementary figure 7 (day 15: ****p < 0.0001, induced hTau-K280R vs. induced hTau-K280Q and vs. induced hTau-wt; two-way ANOVA).

In terms of fly survival, surprisingly, we observed on one hand that both pseudo-acetylation and pseudo-de-acetylation at K280 on hTau reduced fly lifespan to a similar extent (p > 0.05, induced hTau-K280Q vs. induced hTau-K280R, log-rank test, Fig. 8) and on the other hand that both Q and R mutations significantly delayed fly death as compared to the non-mutated hTau-wt (****p < 0.0001, induced hTau-wt vs. induced hTau-K280Q and vs. induced hTau-K280R, log-rank test, Fig. 8).

Altogether, our results indicate that the acetylation status at K280 regulated hTau phosphorylation and protein levels, while the adult-onset neuronal expression of K280Q pseudo-acetylated hTau species led to exacerbated neurotoxicity and fly locomotion defects, an effect that was however not translated into decreased fly survival.

## Discussion

Post-translationally modified hTau proteins are the main constituents of intraneuronal aggregates observed in AD brains. While hTau hyperphosphorylation has extensively been shown to contribute to hTau toxicity (for review see^22^), recent reports implicate acetylated hTau species in the pathological events leading to neurodegeneration in AD^2^,^3^,^5^,^7^,^8^. However, to date, no clear link has been established between acetylated hTau at K280 and toxicity *in vivo*. Using inducible transgenic *Drosophila* lines expressing either acetylation- or anti-acetylation- mimics at residue 280 of the full-length hTau protein, we demonstrated in the present study that hTau-K280Q neuronal expression is detrimental in adult *Drosophila*, exacerbating photoreceptor neuronal degeneration and locomotion defects while modulating hTau levels and phosphorylation.

Transgenic fly lines were generated using the attP/attB targeted integration system, which was previously shown to induce expression of transgenes at comparable levels among the investigated lines (12,19 and Fig. 2). Combining these transgenes with the inducible GeneSwitch system allowed us to bypass potential developmental effects of hTau ectopic expression (for review see^23^) and therefore analyse toxic effects exclusively related to hTau expression in the adult nervous system. Under these conditions, we could observe that the adult-onset expression of the wild-type 2N4R isoform of human Tau in the *Drosophila* nervous system was highly detrimental, leading to strikingly impaired fly climbing ability and survival, and triggering photoreceptor neurodegeneration. While previous studies have reported reduced fly lifespan upon constitutively over-expressed wild-type hTau^13^,^24^, we highlight in the present study drastic toxic effects that are specifically attributable to the adult-onset expression of the longest CNS hTau isoform in the fly nervous system.

To investigate the toxicity induced by K280-hTau acetylation in particular, we used acetylation- and anti-acetylation-mimic mutations^2^,^5^,^15^,^16^,^17^,^18^. Rather than making use of lysine acetyltransferase enzymes such as CBP or p300 to increase overall acetylation of hTau, our site-specific approach allowed us to analyse the specific effects of a single pseudo-acetylated hTau residue *in vivo*. Noteworthy, mimicking lysine acetylation with glutamine or de-acetylation with arginine, respectively, is particularly suitable to investigate the effect of a modified charge.

In this study, we chose to analyse the effects of K280 acetylation on hTau toxicity. Indeed, hTau K280 residue, and more specifically its deletion, was shown to be involved in the pathogenesis of Tauopathies, both in patients and in transgenic models^10^,^25^,^26^,^27^,^28^. Importantly, acetylated-K280-hTau species have been observed within hTau deposits in AD brains^2^,^7^,^8^ and were retrieved in insoluble proteins fractions from brains of AD transgenic mice^2^, altogether supporting a potential role for acetylated-K280-hTau in AD pathogenesis.

Previous *in vitro* reports have shown a regulation of hTau aggregation following multi-acetylation by acetyltransferase enzymes, pointing to either increased^2^ or decreased^4^,^6^ aggregation of hTau. Interestingly, even though we observed lower levels of total hTau proteins in the fly nervous system upon pseudo-de-acetylation of the single K280-hTau residue, we could not highlight any significant change in hTau solubility among the investigated hTau transgenic lines. Our results therefore suggest that modifying the single K280-hTau epitope using acetylation mimics is not sufficient to change hTau aggregation in a way that is detectable *in vivo*. Interestingly, this further implies that acetylation and deletion of K280 follow different pathways to eventually trigger neurotoxicity, as K280 deletion was previously shown to promote hTau aggregation into paired helical filaments^9^. As our system ensured comparable expression levels of transgenes (Fig. 2), the differential hTau protein levels we observed might rather be the consequence of either increased hTau oligomerisation^5^,^21^ or differential protein degradation, involving either the ubiquitin-proteasome system and/or lysosome-mediated autophagy. Indeed, a recent study investigating the consequences of K174 acetylation on hTau-induced toxicity, has suggested that the acetyl-mimic K174Q mutation both increased the rate of hTau oligomerisation together with increasing hTau stability in cell culture^5^. Importantly, we could not detect any specific signal for hTau oligomers in our hTau transgenic lines compared to non-induced controls, suggesting that further experiments will be necessary to determine whether hTau oligomers can be detected in transgenic *Drosophila*. Yet, interestingly, it appeared that hTau clearance was differentially regulated in our hTau transgenic flies. Indeed, by means of a switch on/switch off experiment, we observed that the clearance of hTau proteins in hTau-K280Q flies was significantly delayed compared to that of hTau-wt and hTau-K280R transgenics. These results suggest that the higher induced neurotoxicity and increased protein stability of hTau-K280Q species might be a consequence of their impaired clearance. Though we could not highlight any regulation in the levels of the Hsc70 and HSP90 chaperone proteins, further investigations will be needed to determine how the cell degradation machinery is involved in the regulation of the observed differential hTau clearance.

Next, our results support previous findings suggesting a crosstalk between hTau acetylation and phosphorylation^4^ and give important insights suggesting that hTau post-translational modifications may act in concert *in vivo*. Indeed, we observed higher phosphorylation on Serine 262 and lower phosphorylation on Threonine 212/Serine 214 (AT100), whereas no change was measured for Serine202/Threonine 205 phosphorylation (AT8), in K280Q-hTau mutants as compared to K280R-hTau transgenics, when normalised to total hTau levels. This effect was observed in both 14-day-old and 21-day-old flies. Noteworthy, our results indicated that AT100 phosphorylation levels changed in our hTau transgenic flies without any overt modulation of hTau solubility. This observation is in line with a previous study reporting hTau phosphorylation on the AT100 epitope independently from hTau aggregation^29^.

Regarding hTau-wt species, it is interesting to note that its phosphorylation on S262 and T212/S214 is strictly following neither that of K280Q- nor of K280R-mutated forms of hTau, but rather is a mixture of both. Importantly, this effect is likely to be the specific consequence of the post-translationally editable lysine 280 present in hTau-wt species. Human Tau phosphorylation is a tightly regulated process that underlies both physiological and pathological functions^30^,^31^. It is possible that the acetylation state of K280 regulates hTau conformation, thereby changing the accessibility of some hTau epitopes to kinases and phosphatases, or that it triggers differential signalling pathways leading to different sub-cellular localisations of these hTau species, with a differential enzymatic environment. Altogether, our results suggest that the single K280Q acetylation-mimic mutation is sufficient to induce a significant change in hTau protein phosphorylation, potentially contributing to hTau-induced neurotoxicity.

Finally, our results indicate that despite an exacerbated toxicity of K280Q-hTau on neuronal degeneration and fly climbing ability, expression of these hTau species did not reduce fly survival as compared to hTau-K280R transgenics. Indeed, unexpectedly, we observed that acetylation-mimic and de-acetylation-mimic mutations of K280-hTau triggered comparable toxic effects on fly survival. This points to an uncoupling of the extent of toxicity that is observed on neuronal degeneration, behaviour and fly lifespan. It could be speculated that the involved neuronal subtypes differ in their susceptibility to hTau-induced toxicity, or that fly survival is also regulated by additional factors than the sole neuronal fitness. Indeed, next to neurons, other cell types might be important and involved in the regulation of fly lifespan, as was suggested by a previous report investigating the toxicity of hTau proteins on fly survival when specifically expressed in *Drosophila* glial cells^32^. In the context of the growing evidence that hTau proteins can be secreted by neurons^33^,^34^,^35^, one could speculate that the surrounding glial cells differentially respond to different secreted hTau species, altogether differentially regulating fly lifespan.

Importantly, the alteration of fly survival induced by both K280Q- and K280R-hTau was significantly milder than that induced by the non-mutated hTau-wt species. While further experiments would be required to decipher this issue, one can hypothesize that in hTau-wt transgenic flies, the free lysine residue at position 280 undergoes various conditions that may result in potent toxicity on fly survival. First, in contrast to the K280Q and K280R mutants, hTau-wt flies most likely express two pools of hTau species with regards to K280 acetylation, the stoichiometry of which can be hardly controlled in our system. In addition, the relative abundance of acetylated K280-hTau species may be regulated with age in the *Drosophila* nervous system. Finally, the occurrence of other post-translational modifications at K280 such as methylation or ubiquitination cannot be ruled out in our hTau-wt transgenic line, and this may as well regulate hTau-wt toxicity.

In summary, our results indicate that modulating hTau acetylation at lysine 280 is sufficient to influence hTau neurotoxicity *in vivo*, which, in the context of the massive neuronal loss observed in AD brains, suggests that acetylated K280-hTau species likely contribute to the pathological events leading to neurodegeneration in AD and represent a relevant target for treatment.

## Materials and Methods

### Fly stocks and fly maintenance

All fly stocks were kept at 25 °C or 29 °C on a 12:12 h light:dark cycle at constant humidity and fed with standard sugar/yeast/agar (SYA) medium (15 g L^−1^ agar, 50 g L^−1^ sugar, 100 g L^−1^ yeast, 30 mL L^−1^ nipagin and 3 mL L^−1^ propionic acid). All lines were backcrossed into a white Dahomey (w^Dah^) wild-type, outbred genetic background for at least six generations prior to experiments. The inducible and neuron-specific gene-switch Elav-Gal4 driver line (elavGS) was derived from the original elavGS 301.2 line^36^ and obtained as a generous gift from Dr. Hervé Tricoire (CNRS, France). Adult-onset transgene expression using the elavGS driver was achieved through addition of the activator RU486 (Mifepristone) to fly food at a final concentration of 200 μM. Non-induced controls were obtained by adding the vehicle (i.e. ethanol) to fly food. All elavGS-driven experimental flies were kept at 25 °C throughout development and during the 48-h mating step following eclosion, after which females were sorted and transferred to 29 °C, apart from the lifespan experiment shown in supplementary figure 2b, which was performed at 25 °C.

### Generation of human Tau transgenic fly lines

cDNA from the 2N4R human Tau isoform (441 amino acids, the largest isoform in human central nervous system) was obtained from Gustke et *al.*^37^. Specific mutation of lysine 280 of hTau was achieved using the QuikChange II Site-Directed Mutagenesis Kit following manufacturer’s instructions (Agilent Technologies). hTau constructs were subsequently cloned into the pUASTattB vector, enabling the use of the attP/attB targeted integration system combined with the φC31 integrase to generate transgenic fly lines. Transgenes were inserted into the attP40 landing-site locus to ensure both standard levels of mRNA expression and the best ratio of induced to basal expression^19^. The correctness of hTau sequences was verified in transgenic fly lines by genomic DNA sequencing. All experimental lines were homozygous for the hTau transgene except for supplementary figure 2 where they were heterozygous.

### Lifespan analysis

For lifespan experiments, 150 to 200 once-mated females per group were allocated to vials at a density of 10 flies per vial and subsequently kept at 29 °C or 25 °C. Every 2 to 3 days, flies were transferred to fresh food and the number of dead flies was recorded. Results are expressed as the proportion of survivors ±95% confidence interval.

### Climbing assay

Fly climbing ability was measured and analysed as previously described^12^,^38^ using a countercurrent apparatus. At least 3 replicates of 20 female flies per group were analysed blindly in 3 independent experiments.

### Rhabdomere assay

We used the cornea neutralization technique^20^ to visualize the rhabdomeres from the ommatidia of the fly compound eye. Briefly, dissected fly heads were mounted on microscope slides using nail polish and further covered with oil. The number of ommatidia lacking rhabdomeres was counted using a Leica DMI4000B/DFC 340FX inverted microscope and a 40 × oil immersion objective. At least 50 ommatidia per fly and 5 flies per genotype were examined blindly for each time point.

### Protein sample preparation

20 female heads or 10 third-instar L3 larvae per biological replicate were homogenized by sonication in 200 μL of ice-cold RIPA-1% SDS buffer supplemented with Complete mini without EDTA protease inhibitor (Roche). Protein concentration was measured using the BCA protein assay kit (Pierce) according to the manufacturer’s instructions. 5 to 10 μg of total proteins were supplemented with 2x LDS containing reducing agent (Invitrogen) and heated at 98 °C for 10 minutes prior to western blot analysis.

### Soluble-insoluble fractionation

This procedure was based on Fatouros et *al.* with some modifications^25^. Briefly, heads of 14-day-old females (20 flies per biological replicate) were homogenized by sonication in 150 μL ice-cold RAB buffer (Pierce) and Complete mini without EDTA protease inhibitor (Roche). Following centrifugation at 100,000 g for 1 h at 4 °C, the supernatant (“soluble fraction”) was collected and the pellet was homogenized in 150 μL of RIPA-1% SDS by pipetting. Samples were centrifuged again at 100,000 g for 1 h at RT and the supernatant was collected (“insoluble fraction”). The protein concentration was measured in the soluble fraction using the BCA protein assay kit (Pierce). 5 μg of the soluble fraction and twice the amount of the equivalent volume of the insoluble fraction were used for western blotting.

The western blots displayed in Fig. 5 are representative of levels observed in both soluble and insoluble protein fractions at an exposure time that allows quantification. Noteworthy, these western blots do not reflect the relative proportion of soluble and insoluble hTau species. Such information is provided in Supplementary Figure 4.

### Western blotting

Protein samples were separated on Any kD Criterion gels (Biorad) and subsequently transferred to 0.45 μm nitrocellulose membranes (GE Healthcare). Membranes were blocked in TNT buffer (Tris–HCl 15 mM pH 8, NaCl 140 mM, 0.05% Tween) with or without 5% non-fat dry milk for 1 h at room temperature and incubated overnight at 4 °C with the following primary antibodies: pS202/T205-hTau (AT8, 1/2000, Thermo Scientific), pT212/S214-hTau (AT100, 1/2000, Thermo Scientific), pS262-hTau (1/5000, Invitrogen), T22 (1/1000, Millipore), total hTau K9JA (1/100,000, Dako), total hTau HT7 (1/2000, Thermo Scientific), Hsc70 (HSPA8, 1/1000, Cell signaling), HSP90 (1/1000, Cell signaling), α-tubulin (11H10, 1/2000, Cell Signaling) and β-actin (1/200,000, Abcam). HRP-conjugated anti-mouse or anti-rabbit antibodies (1/10,000, Invitrogen) were used for 1 h at room temperature and detection was performed using ECL chemiluminescence kits (GE Healthcare) and Hyperfilms (GE Healthcare). Bands were quantified using the ImageJ software (Scion Software) and results are expressed as mean ± sem.

### RNA extraction and qRT-PCR

Total RNA was extracted from 1- and 5-day-old female flies (20–25 heads per replicate) using a Trizol-Chloroform-based procedure (Invitrogen) and subsequently treated with DNAse I (Ambion). 300 ng of RNA were then subjected to cDNA synthesis using the SuperScript Vilo Mastermix (Invitrogen). Quantitative real-time PCR was performed using TaqMan primers (Applied Biosystems) in a 7900HT real-time PCR system (Applied Biosystems). Actin5c was used as a normalization control and the relative expression of hTau was determined by the ΔΔ*C*_T_ method. Five to six independent biological replicates per group were analysed. Results are expressed as a percentage of the hTau-wt transgenic line and are plotted as mean ± sem.

### Statistical analysis

For lifespan experiments, statistical differences were assessed using the log-rank test. Other results are expressed as mean ± sem and differences between mean values were determined using either Student’s *t* test, one-way ANOVA followed by Tukey’s post hoc test or two-way ANOVA followed by Tukey’s post hoc test using Graphpad Prism software. *p* values <0.05 were considered significant.

## Additional Information

**How to cite this article**: Gorsky, M. K. *et al*. Acetylation mimic of lysine 280 exacerbates human Tau neurotoxicity *in vivo*. *Sci. Rep.* **6**, 22685; doi: 10.1038/srep22685 (2016).

## Supplementary Material

Supplementary Information
