# Supplementary material for: Acetylation mimic of lysine 280 exacerbates human Tau neurotoxicity in vivo
Source: Sci Rep. 2016 Mar 4;6:22685. doi: 10.1038/srep22685 (PMC4778021; doi:10.1038/srep22685)

SUPPLEMENTARY INFORMATION

Acetylation mimic of lysine 280 exacerbates human Tau neurotoxicity in vivo

Marianna Karina Gorsky[1], Sylvie Burnouf[1], Jacqueline Dols[1], Eckhard Mandelkow[2,3] and Linda Partridge[1]*

[1]Max Planck Institute for Biology of Ageing, Joseph-Stelzmann-Strasse 9b, 50931 Cologne, Germany; CECAD Cologne Excellence Cluster on Cellular Stress Responses in Aging Associated Diseases, 50931 Cologne, Germany.
[2]German Center for Neurodegenerative Diseases (DZNE), 53175 Bonn, Germany; CAESAR Research Center, Ludwig-Erhard-Allee 2, 53175 Bonn, Germany.
[3]Max-Planck-Institute for Metabolism Research, Hamburg Outstation, c/o DESY, Hamburg.

*Correspondence should be addressed to L. P. (Linda.Partridge@age.mpg.de; Telephone: 0049(0)221-37970602; Fax: 0049(0)221-3797088602).

SUPPLEMENTARY FIGURE LEGENDS

Supplementary Figure 1. The inducible gene switch system. Total hTau levels were measured during development (third-instar L3 larvae) and adulthood (14 days old) in elavGS-driven UAS-hTau-wt transgenic flies exposed ("RU-induced") or not ("non-induced") during 14 days to the RU486 inducer. The levels of hTau retrieved from hTau-wt larvae and non-induced adult flies were negligible compared to those of RU-induced hTau-wt flies. Extracts from elavGS adult flies were examined as a negative control for hTau detection. The Actin signal confirmed comparable protein amounts among the samples.

Supplementary Figure 2. Lifespan analysis of heterozygous hTau-wt transgenic flies. The survival of transgenic flies expressing one copy of the hTau-wt transgene (elavGS-driven) was evaluated at 29˚C (**a**) and 25˚C (**b**). While hTau-expressing flies (plain line) showed a reduction of median lifespan of 15% compared to their non-induced controls (dotted line) at 29˚C ($p<0.0001$, log-rank test), no significant difference could be observed between induced and non-induced transgenics at 25˚C ($p>0.05$, log-rank test).

Supplementary Figure 3. hTau phosphorylation and total levels in 21-day-old flies. Western blot analysis **(a)** and quantification **(b)** of hTau phosphorylation on S262 and T212/S214 (AT100) and total hTau levels using the polyclonal K9JA (Dako), following 21 days of hTau expression in the fly nervous system using the elavGS driver. Results are normalised to total hTau levels and to Actin, and expressed relative to levels observed in the hTau-wt transgenic line (*$p<0.05$ and **$p<0.01$, one-way ANOVA followed by Tukey's post hoc test, n=5/genotype).

Supplementary Figure 4. hTau in *Drosophila* is highly soluble. Representative western blot of the relative proportion of soluble and insoluble hTau species

retrieved following fractionation of protein extracts from hTau-wt, hTau-K280Q and hTau-K280R transgenic *Drosophila* heads.

Supplementary Figure 5. Evaluation of hTau oligomerisation. Western blot analysis of hTau oligomerisation in 14-day-old hTau-wt, hTau-K280Q and hTau-K280R fly heads (elavGS-driven) using the T22 oligomer-specific antibody did not highlight any specific band for hTau oligomers compared to their age-matched elavGS driver control.

Supplementary Figure 6. Evaluation of Hsc70 and HSP90 chaperone levels. Western blot analysis (**a**) and quantification (**b**) of Hsc70 and HSP90 protein levels retrieved from adult fly head following 21 days of hTau-wt, hTau-K280Q or hTau-K280R expression in the fly nervous system using the elavGS driver ($p>0.05$, one-way ANOVA followed by Tukey's post hoc test, n=5/genotype).

Supplementary Figure 7. Beneficial effects of the K280R mutation on hTau-induced toxicity were consistently observed. Example of a replicated climbing assay that was performed over time with flies over-expressing either hTau-wt (red), hTau-K280Q (grey) or hTau-K280R (blue) in the adult nervous system (elavGS-driven). Day 15: ****$p<0.0001$, RU486-induced hTau-K280R vs. RU486-induced hTau-K280Q and vs. RU486-induced hTau-wt; *$p<0.05$, non-induced hTau-K280Q vs. non-induced hTau-wt and vs. non-induced hTau-K280R; two-way ANOVA followed by Tukey's post hoc test.

Supplementary Figure 1 – The inducible gene switch system

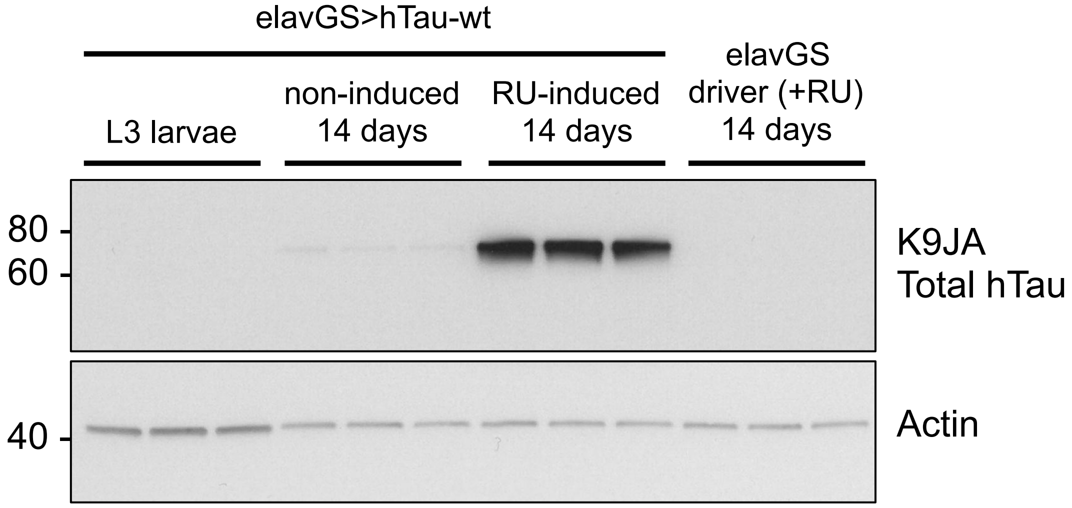

Supplementary Figure 2 – Lifespan analysis of heterozygous hTau-wt transgenic flies

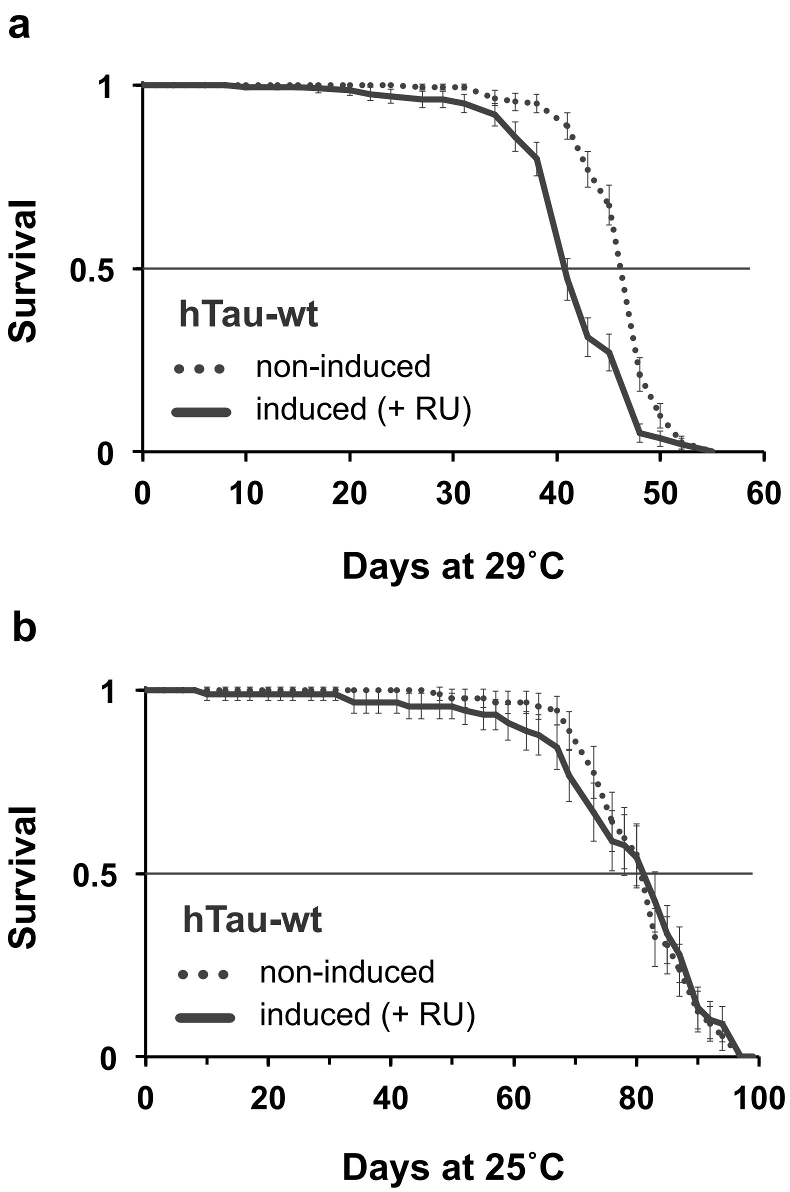

Supplementary Figure 3 – hTau phosphorylation and total levels in 21-day-old flies.

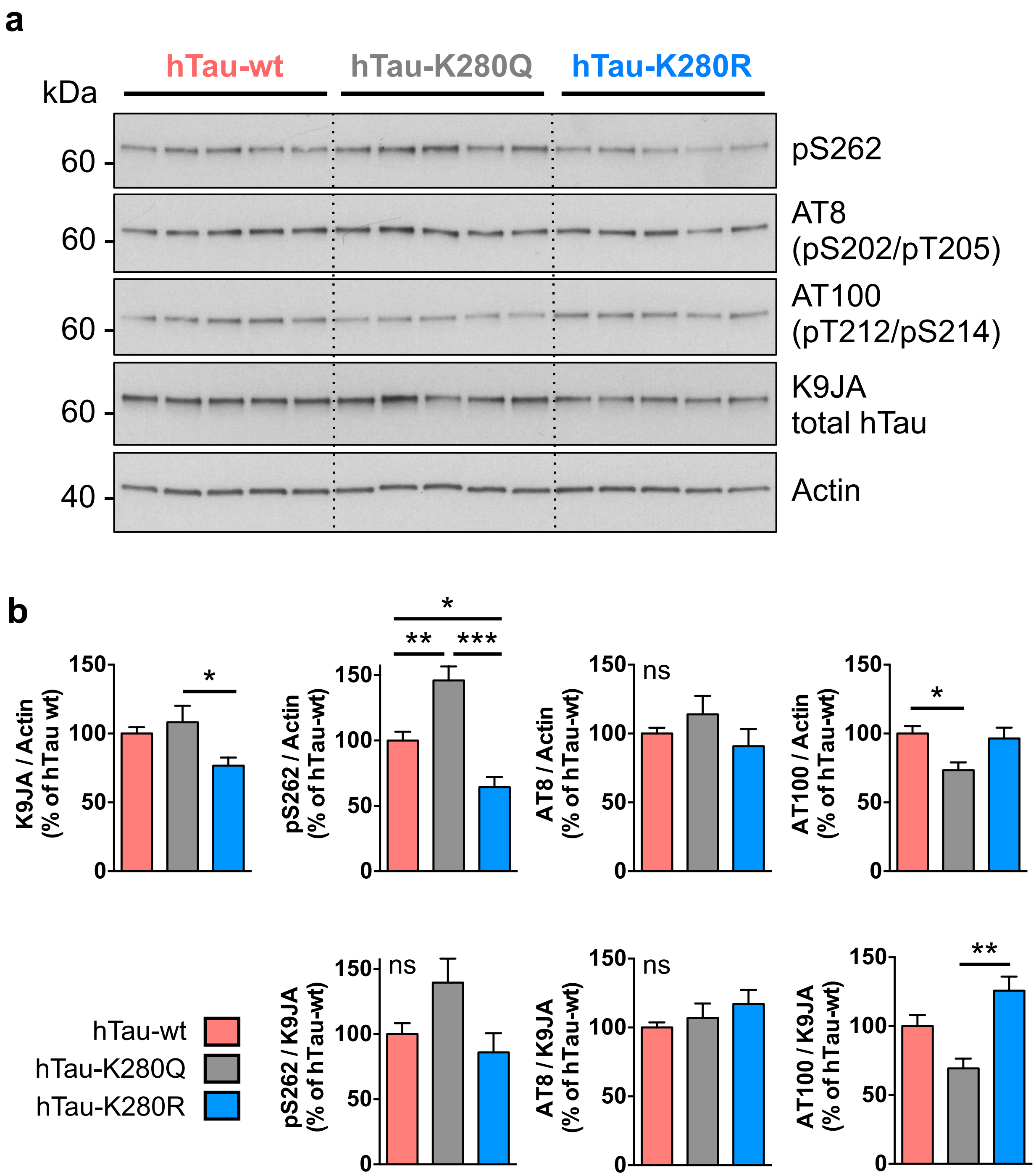

Supplementary Figure 4 – hTau in *Drosophila* is highly soluble

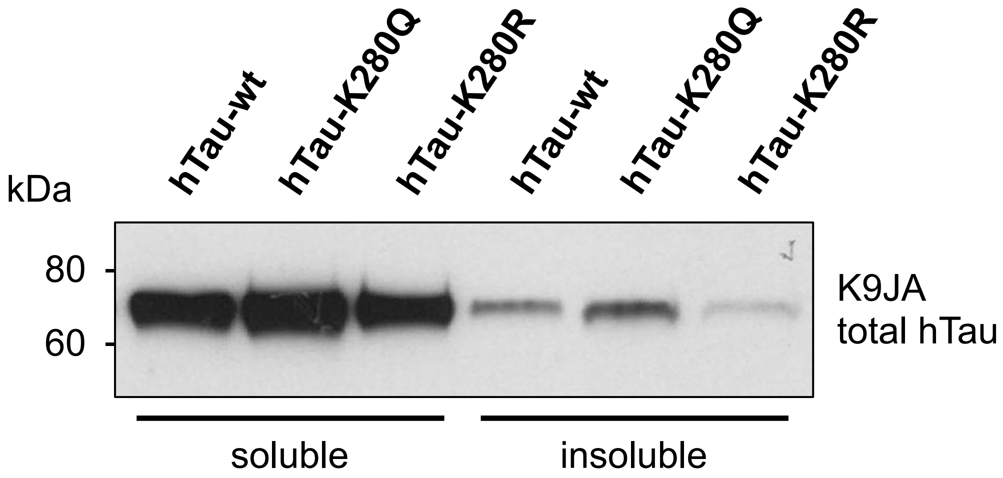

Supplementary Figure 5 – Evaluation of hTau oligomerisation

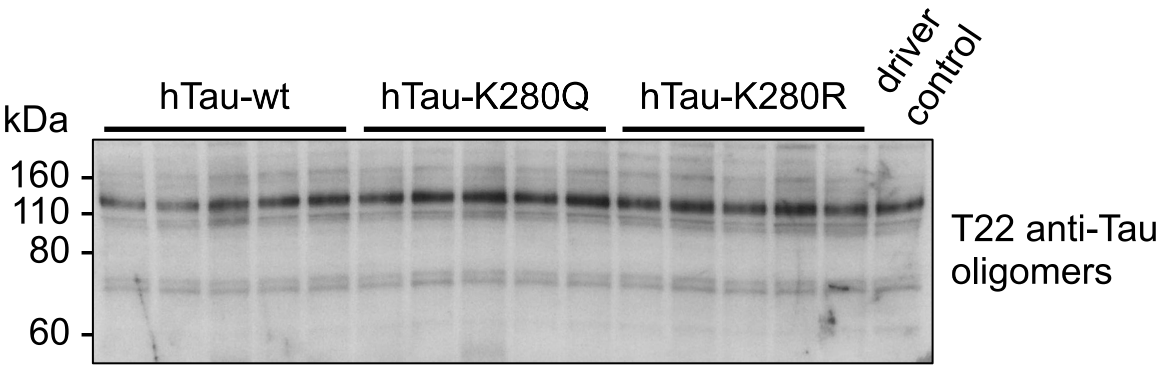

Supplementary Figure 6 – Evaluation of Hsc70 and HSP90 chaperone levels

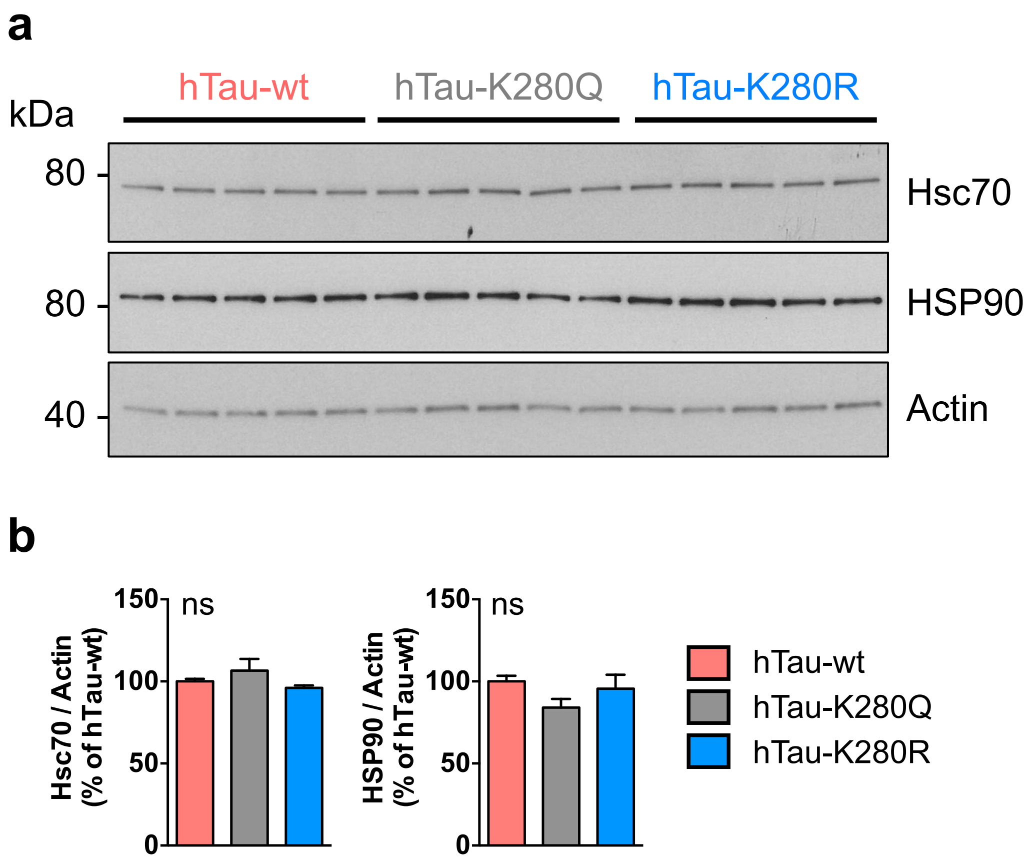

Supplementary Figure 7 – Beneficial effects of the K280R mutation on hTau-induced toxicity were consistently observed.

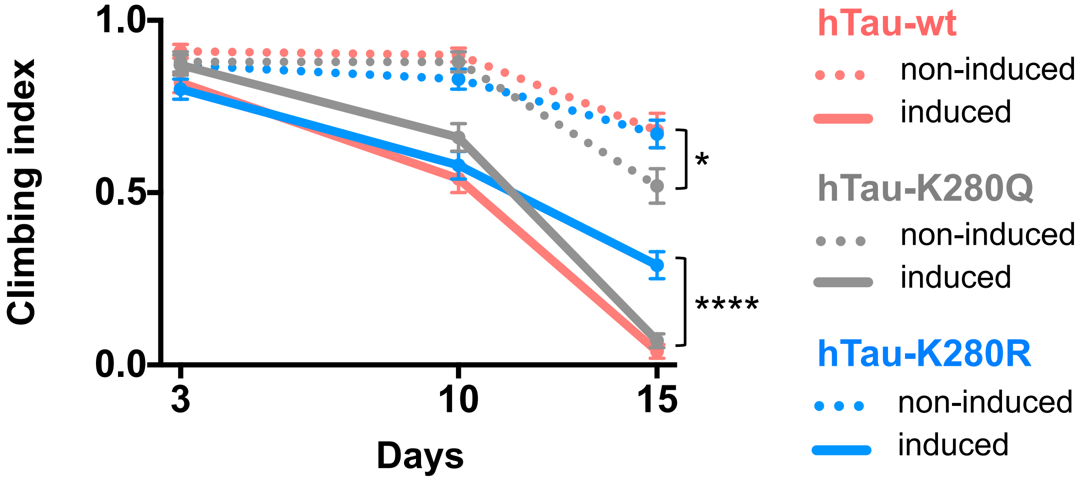

Supplement: Supplementary Information [file srep22685-s1.pdf]
